# Lime as an Anti-Plasticizer for Self-Compacting Clay Concrete

**DOI:** 10.3390/ma9050330

**Published:** 2016-04-29

**Authors:** Gnanli Landrou, Coralie Brumaud, Frank Winnefeld, Robert J. Flatt, Guillaume Habert

**Affiliations:** 1 Institute of Construction and Infrastructure Management, Chair of Sustainable Construction, Swiss Federal Institute of Technology (ETH Zurich), Stefano-Franscini-Platz 5, Zürich 8093, Switzerland; landrou@ibi.baug.ethz.ch (G.L.); habert@ibi.baug.ethz.ch (G.H.); 2 Laboratory for Concrete/Construction Chemistry, Empa, Swiss Federal Laboratories for Material Science and Technology, Überlandstrasse 129, Dübendorf 8600, Switzerland; Frank.Winnefeld@empa.ch; 3 Institute for Building Materials, Physical Chemistry of Building Materials, Swiss Federal Institute of Technology (ETH Zurich), Stefano-Franscini-Platz 3, Zürich 8093, Switzerland; flattr@ethz.ch

**Keywords:** clay, C–S–H, rheology, environmentally friendly materials

## Abstract

This paper focuses on the modification of clay properties with inorganic additives to deflocculate and flocculate inorganic soil for the development of a material that would be as easy to use as the current concrete products, but with a much lower environmental impact. Considering that the rheological behaviour of clays is controlled by their surface charge, we first introduce potential determining ions to deflocculate the clay particles and to reduce the yield stress of the earth material. Their efficiency is characterized using zeta potential measurements and rheological tests. We then achieve the flocculation of clay particles by using natural minerals that slowly dissolve in the interstitial liquid and ultimately precipitate calcium silicate hydrate (C–S–H). The precipitation products are identified by X-ray diffraction and the consequences of this delayed precipitation are followed by oscillatory rheometric measurements. Finally, it is suggested that in this process, C–S–H precipitation is not used as a binding vector but as an anti-plasticizer that removes the inorganic dispersant additives.

## 1. Introduction

Traces of earthen architecture date to 10,000 years ago, and this building material technique is still used in most climates and societies [1]. Without transport and with infinite recycling possibilities, earth is among the building materials which have the lowest environmental impact [2,3] and very efficient temperature and moisture regulation properties for indoor living spaces [4]. Earth construction is currently under strong development, likely due to environmental concerns. However, this development is limited because the conventional earth construction techniques are both time-consuming and labour-intensive. In contrast, cement is an incredibly easy-to-use material but has a significant environmental impact [5]. Substantial engineering and scientific efforts have been invested to improve the understanding and processing of cement-based concrete, but no or very little engineering improvement has been made in the case of earth.

Recent attempts have been made to fluidify earth material and cast it using the same techniques that are used for concrete. Actually, experiments were conducted with castable earth in which cement plasticizers are used to reduce the yield stress at the fresh state and 5 to 8 wt % of cement is added to allow setting [6]. The technique works, but the mechanisms controlling clay behaviour are not well understood, and a large amount of cement is still used. In another experiment of castable earth [7] the product developed is able to flow and set. However, the amount of lime that has to be used is close to 10 wt %, which is a reduction of only approximately 30% compared with conventional cement-based concrete for a compressive strength that does not exceed 15 MPa. Thus, the objective of this study is to use the concrete technologies to transform earthen architecture by providing a material that is as easy and cheap to use as the current concrete products, with the addition of a minimum amount of cement. To achieve this, our approach is to transfer knowledge from concrete technology to earthen construction, recognizing analogies between the two materials. In particular, colloidal interactions and adhesion forces are present in both materials, but there are also differences since the cohesion forces between particles are much weaker for clay particles [8] due to the difference in the constituted binder (no hydraulic reaction occurs).

To improve the material workability, a careful control of the rheology of the clays requires a better understanding of the colloidal interactions between particles [9,10,11] and knowledge transfer from the fundamental physics of grains and colloids to civil engineering [12,13].

Clay, a phyllosilicate mineral based on an octahedral (O) and tetrahedral (T) sheet combine in either a 1:1 or 1:2, respectively, to form an anisotropic TO or TOT layer [14] is an inorganic charged particle. It shows a lamellar morphology with a permanent negative charge at the surface and pH-dependent positive charge at the edge. As a unique binder in earth, clay can have its surface interaction changed with the help of organic dispersants from the cement and ceramic industries. Among the deflocculants commonly used in ceramic processing, sodium hexametaphosphate, a hexamer of composition (NaPO_3_)_6_, guarantees an efficient dispersion effect for natural clay materials [15,16,17,18,19,20,21]. Its deflocculating action is a combination of two mechanisms. First, it increases the overall negative surface charge because of the adsorption of phosphate anions, particularly at the edges of the clay mineral particles, and second by complexing the dissolved flocculant alkaline earth cations and replacing them with Na^+^ cations, thereby increasing the thickness of the electrical double layer [15,16,17,18,19]. The second deflocculant is sodium silicate Na_2_SiO_3_, which is widely used in raw clay and kaolin dispersion as a deflocculant because of its high efficiency [17,18,19,20,21,22,23,24,25,26,27]. Similar to sodium hexametaphosphate, studies have shown that it acts as deflocculant by the adsorption of a polyanion that modifies surface charge as well as by increasing the pH of the solution [22,28]. Other important phenomena, with respect to dispersion stability and rheological behavior, are the dissolution of the clay mineral constituents [29,30,31] and the reactions between the sodium silicates and the dissolved or desorbed ions from the particles [18].

In this paper, we first assess the efficiency of these two clay dispersants and determine how valuable they could be for the development of a self-compacting clay concrete. Then, it is of fundamental importance to be able to flocculate earth material again to remove the formwork and allow for the complete drying of the material. This second stage is currently achieved through the addition of cement, which provides additional strength [1,6,7]. In this study, to develop a material without cement use while transferring technology from cement chemistry and ceramic science, we focus on a different technique and explore the potential role for Ca^2+^ ions coming from the dissolution of natural minerals that contain calcium. This allows for the formation of insoluble salts between the dispersing anions (phosphate and silicate), as well as the restoration of calcium as a flocculating agent between the particles.

## 2. Materials

### 2.1. Earth

**Figure 1:**
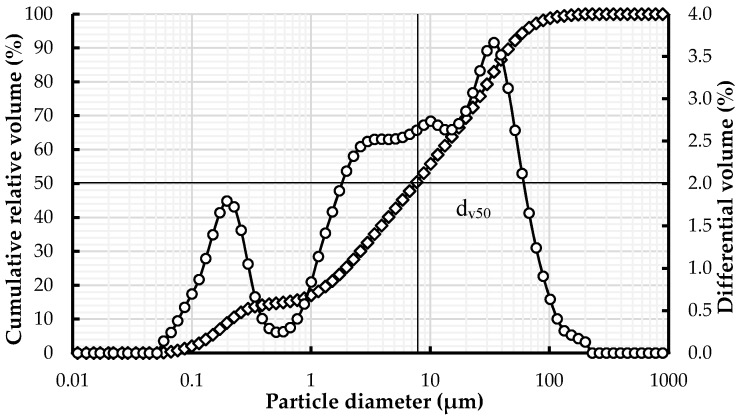
Earth particle size distribution and differential volume.

A commercially available mineral earth material for plastering with a specific density of 2.72 g/cm^3^ and specific surface area *S*_BET_ = 46.9 m^2^/g was used in this study. The particle size distribution (PSD) was measured using a laser particle size analyzer (Partica LA-950, Horiba, Munich, Germany). For the measurements, the earth injected dry in the machine sample bath was dispersed with 0.1 wt % of sodium hexametaphosphate (Fisher Chemical, Reinach, Switzerland). Between each measurement, an automatic ultrasonic treatment in the sample bath was applied during 1 min (repeated 10 times) to avoid agglomeration between particles. The studied clay earth contained 95% fine particles (<63 µm) (Figure 1), including clays (36.6 wt %) and silt (57.9 wt %). Its chemical composition, obtained through X-ray fluorescence spectrometry (XRF), is given in Table 1. The X-ray powder diffraction technique revealed, thanks to Rietveld methods [32,33], that the main mineralogical components are smectite (24 wt %), quartz (41 wt %), a lower amount of kaolinite (7.5 wt %), and traces of carbonate (0.4 wt %). The complete mineralogical composition is given in Table 2. When prepared as a suspension, earth develops a pH of the order of 9.

### 2.2. Dispersants-Deflocculants

Three different dispersants used in the cement and ceramic industries were investigated.

The first one was a high-range water reducer agent (HRWRA), a comb co-polymer that is commonly used in concrete and mortar applications to improve flow material properties by deflocculating cement particles [34]. It is based on a polycarboxylate ether (referred to here as PCE) composed of polyethylene oxide side chains grafted with a methacrylic acid backbone.

The two other dispersants were inorganic deflocculants taken from the ceramic industry: high purity sodium hexametaphosphate (NaPO_3_)_6_ (>99.0% pure) (referred to in this paper as NaHMP) in powder form sourced from Fisher Chemical (Reinach, Switzerland) and sodium silicate solution (noted here NaSil) from Sigma Aldrich (Buchs, Switzerland) with a composition of 10.6% Na_2_O and 26.5% SiO_2_, the rest being water.

All dispersant are added as a solution at the same time as the water.

### 2.3. Calcium Products

Three different calcium products were used as additives: calcium hydroxide (Ca(OH)_2_, >96% pure), calcium carbonate (CaCO_3_, >99.0% pure), and calcium chloride (CaCl_2_, >93% pure), all sourced from Fluka—Sigma Aldrich (Buchs, Switzerland). These products have different Ca^2+^ release rates in the interstitial solution because of their different solubilities.

## 3. Measurement Procedures

### 3.1. Rheology Measurements

Rheology measurements were performed on earth pastes to study the effect of dispersants on the rheological properties of the suspension. To compare the results obtained from different measurements, the water to solid ratio (W/S) for earth pastes was kept constant (0.5). Only the nature and dosage of the dispersant were varied from one sample to another: these variations are discussed in the relevant sections of this manuscript. In this paper, all dosages are expressed as the percentage of the mass of solids in the system, and water is deionized. The tested earth pastes were prepared using the following mixing procedure: the dispersant in powder or liquid form was added to the required amount of mixing water to ensure its dissolution before introduction into the solid phase. Water (with or without dispersant) was mixed with earth for 3 min at 365 rpm with a mechanical stirrer equipped with a four-bladed mixing tool (Heidolph, Dietikon, Switzerland).

#### 3.1.1. Yield Stress Measurements

**Figure 2:**
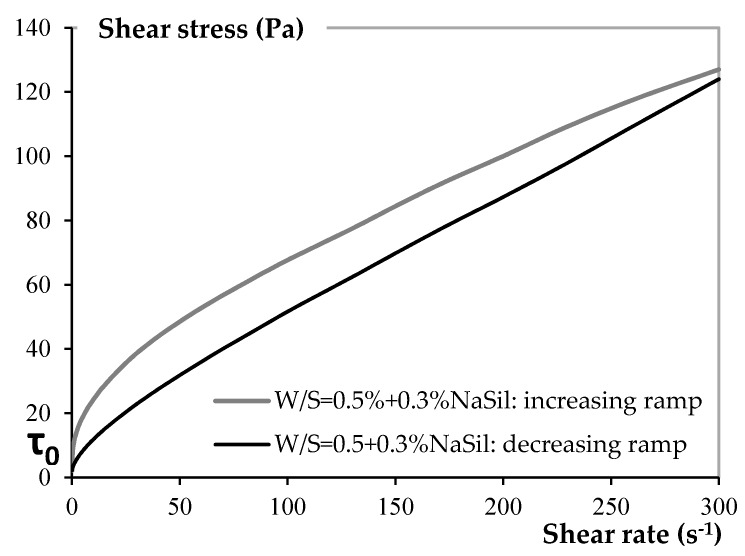
Flow curve of a clay paste prepared with 0.3% of NaSil.

The rheology measurements were performed using a MCR501 Anton Paar (Buchs, Switzerland) stress-controlled rheometer equipped with Vane geometry [35] at room temperature (23 °C ± 0.1 °C). The Vane geometry was a four-bladed paddle with a diameter of 22 mm. The outer cup diameter was 25 mm, and its depth was 60 mm. Twenty minutes after the first contact of the constituents (including the mixing phase), the cup of the rheometer was filled and covered to limit evaporation, and the sequence was started. An increasing shear rate ramp from 0.1 to 300 s^−1^ (with a logarithmic distribution of shear rates) was then applied for 300 s followed by a decreasing shear ramp from 300 to 0.1 s^−1^ for 300 s. Only the decreasing ramps are analyzed for the value of the yield stress (Figure 2), extrapolated at low shear rates according to the Bingham model $\tau =\tau_{0}+\eta_{p}\dot{\gamma }$, where τ is the shear stress, $\dot{\gamma }$ the shear rate, τ_0_ the yield stress and η_p_ the plastic viscosity.

#### 3.1.2. Viscoelastic Properties

The limits of the linear viscoelastic behavior (LVR), where the rheological properties of the materials are not strain- or stress-dependent, were first determined by identifying the critical value of stress τ_c_ through a stress sweep test. A stress amplitude (from 0.1 to 3000 Pa) at a constant frequency of 1 Hz was applied and the end of the linear elastic regime was defined as when the elastic modulus falls to 90% of the plateau value [36]. The dynamic viscoelastic properties of the materials can be quantified in terms of the storage modulus *G*’ within the linear viscoelastic region (LVR). The viscoelastic behaviour of the pastes was thus investigated in the time domain by applying for six hours a constant shear stress of 1 Pa (lower than τ_c_) and a constant frequency of 1 Hz to the material, and by recording the subsequent strain. This test is used as an indication of the time-dependent structural changes within the material.

### 3.2. Zeta Potential Measurements

The ζ-potential, which is the electrokinetic potential in colloidal systems and, thus, the key indicator of the stability of dispersions, of the concentrated suspensions of earth was measured with a ZetaProbe (Colloidal Dynamics, Hofheim, Germany) [37], which is based on the electro-acoustic method. A high-frequency alternating electric field is applied and causes charged particles to oscillate. The motion of the particles generates a sound wave, which is recorded and delivers the dynamic mobility of the suspended particles. The software calculates the ζ-potential from the dynamic mobility [38,39]. The ζ-potential experiments were conducted on suspensions of solid volume fractions of approximately 6 wt %. The sedimentation issues and resulting measurement artefacts are avoided by blade stirring. The dispersant dosages were kept the same as those used in the rheology measurements. The single point measurement performed enables to follow the ζ-potential over time at constant dosage in the mineral soil. The reported value was recorded once no variation is observed in surface charge.

### 3.3. Mineral Characterization

To understand the role played by calcium, the dispersants and the calcium source have been mixed with a magnetic stirrer at 900 rpm. After mixing 3 min or 3 h, the samples are dried at 60 °C and 50 mbar. This drying process preserves the microstructure of weak crystallized reaction products compared with higher temperature oven-drying techniques (classically 105 °C). It is similar to the pre-treatment used to prepare concrete samples for mercury intrusion porosimetry in [40]. The measurement was done without clay as it is assumed that, even if clay particles in the whole system may react in presence of NaHMP or NaSil under alkaline conditions, the studied reaction is the same as the concentration of released ions during the reaction is low in such a diluted system [18]. The impact of the presence or not of clay for the identification of reaction products was thus negligible. The mineralogical composition of the formed reaction products was determined on randomly oriented powder specimens with X-ray diffraction (XRD). A frontloading XRD specimen was prepared using two blades to minimize the preferred orientation. XRD measurements were performed using Bragg–Brentano geometry (Bruker AXS D8 advance, CuKα radiation, Karlsruhe, Germany). The powder samples were step-scanned at room temperature from 2° to 80° 2θ (step width 0.02° 2θ, counting time 4 s). Finally, the reaction products were also observed with an environmental scanning electron microscope (SEM) (FEI quanta 200 3D).

### 3.4. Adsorption Measurements

A total organic carbon (TOC) analyzer was used in this work for samples prepared with PCE, only organic dispersant used in this study. The analysis technique involves a two-stage process commonly referred to as TC–IC. It allows for the measurement of both the amount of inorganic carbon (IC) by acidification of the sample and the amount of total carbon (TC) in the sample. TOC is calculated by subtraction of the IC value from the TC of the sample. A blank value was first done with an earth paste sample not containing any PCE in order to take into account the amount of organic carbon in earth as a reference. Furthermore, another measurement of the organic carbon in a reference solutions of PCE was done. The earth pastes with different amounts of PCE (0%, 0.5%, 1%, and 2%) were prepared according to the same procedure as for rheological measurement (W/S = 0.5). After mixing, the pore solution was collected through a syringe equipped with a nylon filter 0.45 µm by air pressure filtration. The extracted liquid was then tested with the TOC analyzer Sievers 5310C GE Analytical Instruments (Basel, Switzerland). By subtracting the TOC value measured on the extracted interstitial fluid (and corrected from the baseline value of the earth) from the value obtained in the reference PCE solution, the amount of PCE adsorbed or absorbed on earth particles was computed.

## 4. Experimental Results and Discussion

### 4.1. Effect of Dispersants on Earth Products

#### 4.1.1. Rheology

**Figure 3:**
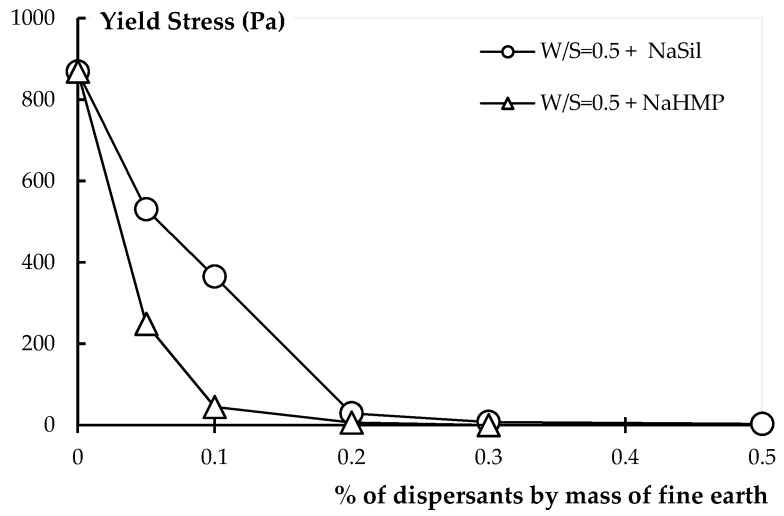
Effect of NaHMP and NaSil on the yield stress of earth paste.

In Figure 3, the yield stress is plotted as a function of dispersant dosage for the earth pastes prepared with NaHMP and NaSil. The water to solid ratio is equal to 0.5, which allows for an initial material that is sufficiently thick to evaluate the efficiency of admixtures. As expected, when the dispersant is added, the yield stress of the earth paste is significantly reduced. The addition of only 0.1% NaHMP significantly decreases the yield stress of the reference paste by a factor of 20. This can be attributed to the deflocculating action of the inorganic dispersant, which modifies the clay surface [16,18,19]. Actually, as the clay’s rheological behavior is controlled by the surface charge, increasing the surface charge with charged inorganic additives deflocculates the clay particles, which considerably reduces the yield stress of the earth material. This observation will be confirmed by ζ-potential measurement in the next section. It can also be noted in Figure 3 that the yield stress becomes constant from 0.2% NaHMP and 0.3% NaSil. We estimates that this is related to the saturation dosage of each dispersant. Beyond these respective dosages, and for this W/S ratio, it is expected that all particle surfaces are covered, and an additional dispersant is no longer efficient. Finally, it is worth noting that the NaHMP deflocculant is more efficient than NaSil, but it is less robust. A small variation of the dosage, which can easily occur in practice, leads to a yield stress close to zero. Since the yield stress of the constitutive paste controls the stability of the material [13,41,42], this very low yield stress could lead to static segregation issues. To prevent these issues, the yield stress of the paste must be sufficiently high to support particles in suspension. According to [42] and considering the maximum particle size of the earth material of 100µm (*cf.* Figure 1), the minimum yield stress needed is of the order of 9 Pa.

**Figure 4:**
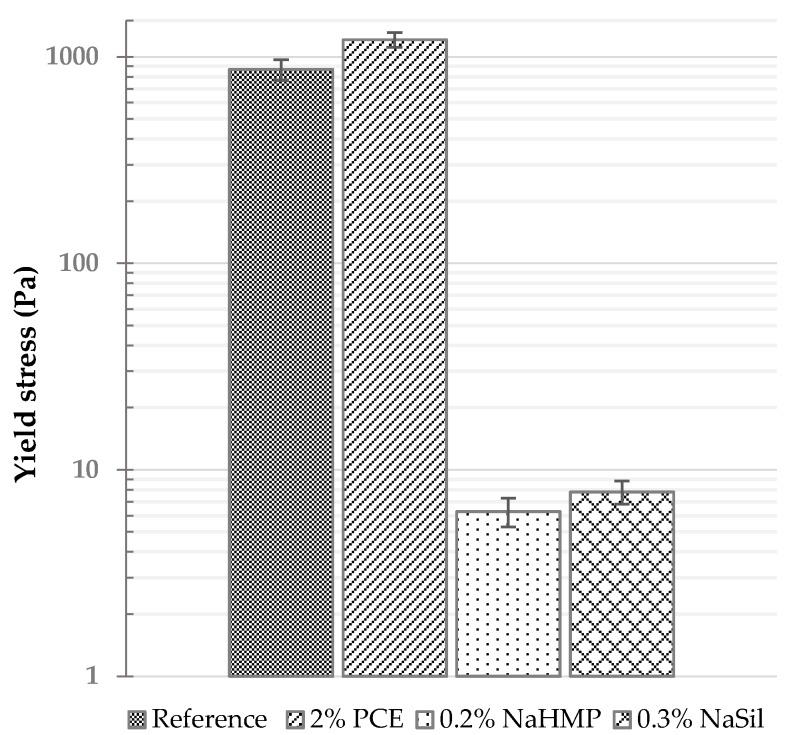
Comparative yield stress of earth pastes mixed with different dispersants.

In Figure 4, earth pastes containing 0.2% NaHMP and 0.3% NaSil are compared with the earth paste containing PCE as dispersant. Adding 2% PCE, which is a high dosage for this product, does not decrease the yield stress of the suspension. On the contrary, a negative effect is observed given that the yield stress of the earth paste prepared with 2% PCE is higher than the yield stress of the reference earth paste. In cement applications, the dispersing effect of PCE is primarily due to adsorption on cement particle surfaces (positively-charged) with a negatively-charged backbone. This induces predominantly steric repulsive forces between particles since cementitious systems have a high ionic strength that basically suppresses electrostatic repulsion [43,44]. As no effect is observed with clay particles, we can suppose that the side chains of PCEs are “trapped” between the aluminosilicate layers of smectite, leading to a large polymer consumption but no dispersion. This phenomenon is well known [45,46,47,48] and will be confirmed with TOC and ζ-potential measurement in the next section.

#### 4.1.2. Zeta Potential

**Figure 5:**
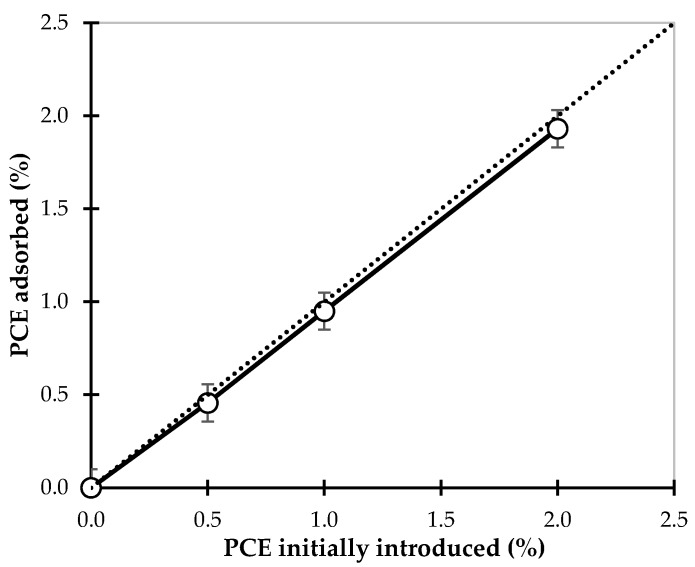
Adsorption isotherm for the PCE molecules reported with respect to the added PCE. The dotted line indicates full consumption.

The ζ-potential provides an indication of the surface charge of the clay particles in suspension. The magnitude of this parameter is often used as a measure of the strength of the repulsive interactions between similarly charged particles in suspensions. The same approach has been applied to evaluate the electrostatic forces between our clay particles. ζ-potential measurements were performed, and the particle charge values for the reference paste and those prepared with different types and amounts of dispersants are gathered in Table 3. In deionized water, the ζ-potential of the earth sample is negative because of its negative surface charge. The addition of 0.2% NaHMP increases its magnitude by a factor close to three. A similar observation can be made for 0.3% NaSil, though this increase has a lower intensity (increase by a factor close to two). These results confirm the interpretation that these two inorganic dispersants deflocculate clay particles by increasing the repulsive forces between particles. NaHMP is more efficient than NaSil in terms of deflocculation, which is correlated with higher negative charge of particles. On the contrary, the ζ-potential of earth material does not change with the addition of PCE, which indicates that the PCE molecules does not affect the surface charges of clay particles. This is no surprise as these polymers are not highly charged, that they are known to induce only minor changes to the zeta potential in addition to dispersing through steric hindrance rather than electrostatic repulsion. More important information with regard to the PCE behavior can be obtained by looking at its adsorption isotherm measured with TOC analysis and plotted with respect to PCE dosage [49]. Indeed, Figure 5 shows that almost 100% of the polymer introduced is adsorbed and that no plateau is observed. This (most probably) indicates that the introduced polymer is captured by the clay minerals with at least some of its side chains being trapped into clay layers, so that its dispersing efficiency is lost. This sorption of extremely large amount of PCEs has been well described in literature for the clay mineral types present in the studied material (smectite) [45,46,47,48]. No saturation of the clay particles is observed which could suggest that at a higher dosage PCEs could be active as dispersants. However, 2% PCE is already a very high dosage which means that reaching the saturation level will not be cost competitive.

### 4.2. Effect of Releasing Calcium

Various types of calcium-containing compounds were introduced into the mixed earth/dispersant to study their effect on the rheology of the material and their potential ability to flocculate particles. The dosages were calculated in order to introduce the same amount of calcium in the system (regardless of solubility). Depending on the dispersant used, the ratios of Ca/Si (when NaSil is used as dispersant) and Ca/P (for NaHMP) were fixed, respectively, at 1.3 and 1.5. Note that these amount are quite small in comparison to earth material as the ratio of 1.3 to 1.5 is calculated in relation to the dispersant which is, itself, calculated in relation to the earth content (0.2 wt %). In comparison to previous studies [6,7], we are then using ten times less calcium salts.

#### 4.2.1. Calcium Ions and Structuration over Time

**Figure 6:**
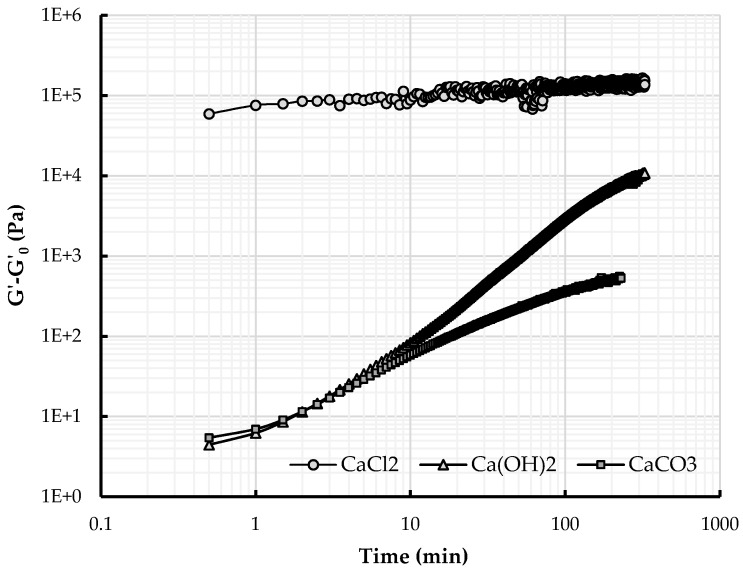
Variation of the storage modulus for earth pastes prepared with 0.2% NaHMP and different calcium products. Values are reported as a difference with respect to *G*_0_, the modulus of the paste without addition of calcium compounds at each measuring time.

**Figure 7:**
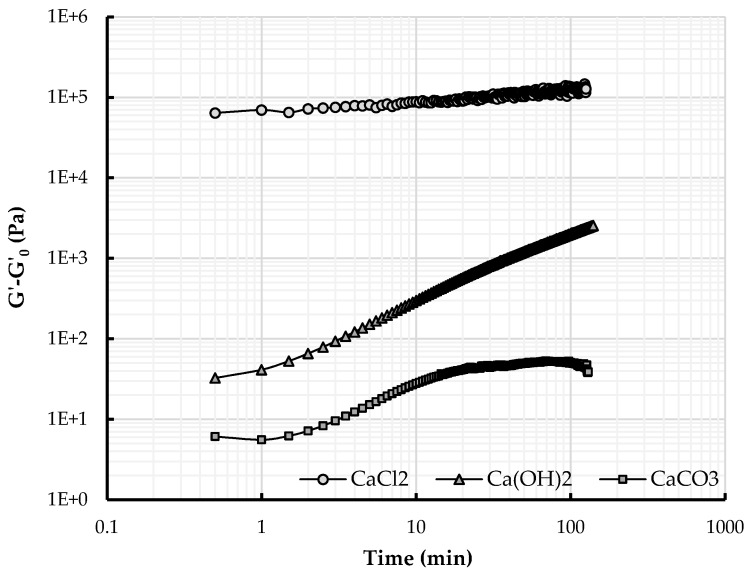
Variation of the storage modulus for earth pastes prepared with 0.3% NaSil and different calcium products. Values are reported as a difference with respect to *G*_0_, the modulus of the paste without addition of calcium compounds at each measuring time.

In Figure 6 and Figure 7, the variation of the storage modulus *G*’ − *G*_0_ (*i.e.*, the storage modulus of the earth paste prepared with calcium and NaSil or NaHMP *G*’ minus the storage modulus of earth paste containing only dispersant *G*_0_) is plotted as a function of time. The elastic modulus is an indicator of the time-dependent structural changes within the material and is an indication of the strength of the particle network at a given time. As a general observation, for all tested earth pastes, the elastic modulus increases with time. This behavior is entirely ascribed to the effect of calcium addition because, by referencing to the neat paste, we account for the potential contribution of water absorption and evaporation that can occur during the test and thereby modify the microstructure. This first observation shows that the use of calcium modifies the internal structure and cohesion of the material. Adding calcium flocculates the system. In the case of NaHMP, some authors have described this phenomenon as a complexation between phosphate anions and calcium ions [16,50] and an interaction of cations with the surface of clay minerals. Andreola *et al.* [15] reports that dissolved Ca^2+^ ions have deleterious effects on dispersing clay materials by (i) adsorbing onto the clay surface, thereby reducing the thickness of the electrical double layer; and (ii) decreasing the amount of phosphate anions that are available to be adsorbed on clay edges through soluble complex formation. By complexation with calcium ions Ca^2+^, a network of interaction is created between particles, which gives rise to cohesion forces within the system. If we consider each earth paste behavior in detail, for the three calcium containing compounds introduced into the earth paste containing 0.2% NaHMP, the evolution is not similar, although the elastic modulus increases with time. First, it can be noted that the earth paste prepared with CaCl_2_ displays a high elastic modulus at the start of the test followed by a low rate of increase (Figure 6). For Ca(OH)_2_ and CaCO_3_, the elastic modulus at the beginning of the test is relatively low and increases rapidly during the test. The effect is more pronounced for the earth paste prepared with Ca(OH)_2_, where the elastic modulus tends to ultimately reach the same high elastic modulus value as the earth paste prepared with CaCl_2_. The same observation can be made for the earth pastes initially prepared with NaSil as deflocculant and calcium (Figure 7), apart from the elastic modulus of the earth paste containing CaCO_3_. The curve can be decomposed in three parts: a plateau is observed at the beginning of the test, followed by an increase and then, after approximately 20 min, a plateau. Considering that the modification of the cohesive network within the material is linked to the interaction between calcium ions and mineral anions (same amount for each set of experiments), the different viscoelastic behaviors can be explained by the accumulated amount of calcium released in solution at a given time or, more accurately, the amount of calcium that has reacted with the mineral anions (Section 4.3). These three calcium products do not present the same dissolution rate, and calcium ions are slowly or quickly released in the interstitial solution depending on the type of calcium containing compound used. The calcium ions coming from CaCl_2_ are released almost instantaneously in the interstitial fluid, which leads to the high initial elastic modulus displayed and, thus, to an initially-structured state, whereas the calcium ions coming from Ca(OH)_2_ are slowly released over time, which delays the material structuration or flocculation. It can be suggested that, in the given time, the total amount of CaCO_3_ cannot be dissolved in the interstitial fluid: the calcium ions coming from this mineral are, thus, not entirely available for the reaction, leading to a slowed down structuration rate.

#### 4.2.2. Structuration and Nature of Interactions

**Figure 8:**
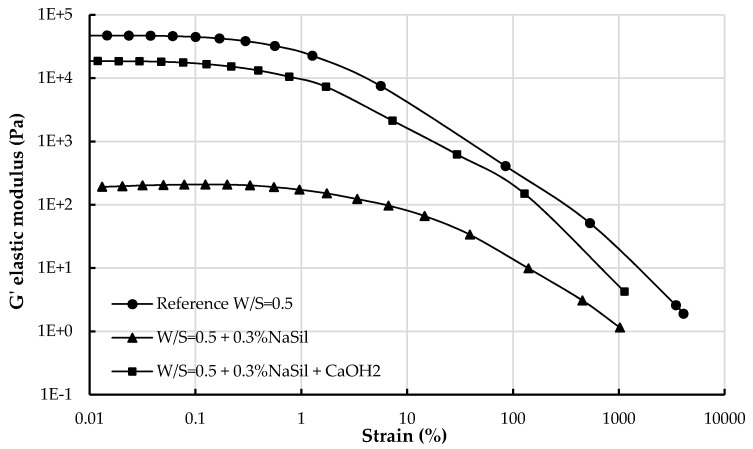
Elastic modulus as a function of strain for the reference earth paste, the earth paste prepared with 0.3% NaSil and the same earth paste containing Ca(OH)_2_ after setting.

In Figure 8, the elastic modulus is plotted as a function of strain for the reference earth paste, the paste prepared with 0.3% NaSil and the same earth paste containing Ca(OH)_2_ obtained from stress sweep test to determine the limit of the elastic linear regime. As previously described, adding 0.3% NaSil decreases the elastic modulus, higher the critical strain of the reference earth paste and modifies the structure of the material by flocculation.

When Ca(OH)_2_ is added to the system, the earth paste presents the same behavior as the reference earth paste: calcium addition therefore allows a return to the initial state and reflocculates the particles. We now focus on the critical strain. When the critical strain is reached, the initial structure of the material has been sufficiently modified to produce the rupture of the particle network of interactions [13,51,52]. The order of magnitude of the critical strain highlights the ability of the structural network to be deformed under stress and defines the nature of interactions. Whereas a small critical strain is associated with short range links between the particles, a large critical strain involves non-contact interactions between the particles [13,53]. In cement suspensions, small critical strain (on the order of 10^−2^%) are characteristic of short range links between C–S–H particles and associated with interaction between crystallised hydrated structures. Larger critical strain (of the order of 1%) can be associated with the breakage of the network of colloidal interactions (*i.e.*, van der Waals attractive forces) between cement particles [13,53].

In our study, the critical strain for the earth paste prepared with 0.3% NaSil is of the order of 10%, whereas the critical strain for the two other earth pastes is of the order of 1%. Our results are consistent with reports in literature. First because NaHMP and NaSil are efficient to disperse clays [15,16,17,18,19,20,21,22,23,24,25,26,27]. Secondly, by comparing these values with those found for cement suspensions [53], it is then possible to suggest that strong but short ranged colloidal interactions are at the origin of the cohesion of the earth paste and not C–S–H links. When 0.3% NaSil is added to the earth material, the critical strain increases, and the material can be even more deformed under stress than the reference paste, indicating that the interaction network between particles is long ranged. This observation suggests that NaSil modifies the van der Waals attractive force network at the origin of this critical strain. As mentioned above, NaSil is able to strongly deflocculate particles in suspension through its adsorption at the surface of the clay particles. We, therefore, suggest that adsorbed NaSil is at the origin of steric repulsive forces between particles, leading to a dispersion of particles into suspension. When Ca(OH)_2_ is added, the critical strain of the earth paste is similar to the critical strain of the reference earth paste: the initial state is recovered. Adding calcium allows then a movement from a material displaying a critical strain of the order of 10% to a material with a critical strain of the order of 1%. The internal structure and the type of interactions of the material are not modified: after deflocculating the particles with the use of a dispersant, they reflocculate under their initial form under the effect of calcium. The fact that we observe an equivalent critical strain leads to the assumption that calcium addition annihilates the plasticizing effect of the dispersant by removing it from the clay surface. The amount of calcium introduced into the mixture corresponds to the Ca/Si ratios that are frequently used to produce synthetic C–S–H. It is therefore most likely that the dispersant has been consumed by the precipitation of C–S–H products into the interstitial solution. However, the amount of C–S–H produced is not sufficient to control the critical strain of the suspension as no inflexion of the curve is observed at 10^−2^. The nature of the precipitation products needs to be identified to support this hypothesis.

### 4.3. Identification of Reaction Products

**Figure 9:**
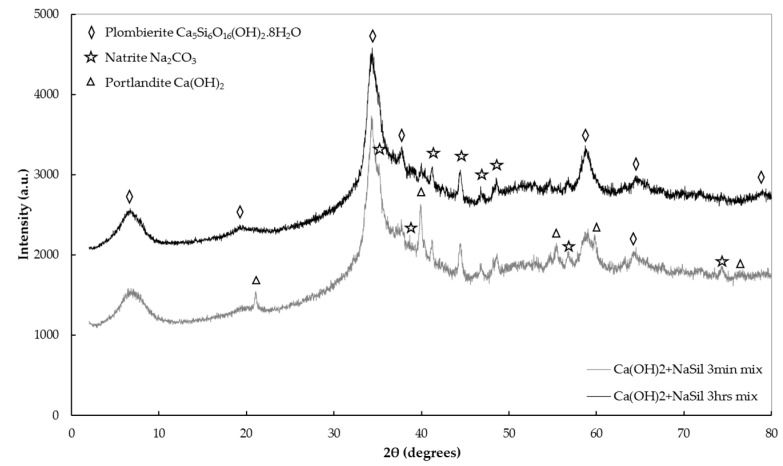
X-ray diffraction patterns of NaSil solution and Ca(OH)_2_ at different mixing times, dried at 60 °C and 50 mbar.

**Figure 10:**
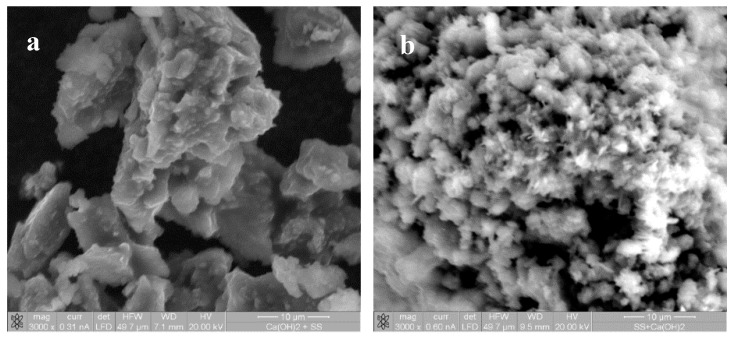
SEM images of NaSil and Ca(OH)_2_ precipitation products dried at 60 °C and 50 mbar after (**a**) 3 min; and (**b**) 3 h of mixing.

The reaction products have been identified through XRD and SEM on samples where only the dispersant and the calcium source have been mixed. When the NaSil is used as a dispersant, a fast disappearance of Ca(OH)_2_ peaks (still present after three minutes but absent after three hours) and the precipitation of two phases are observed: formation of plombierite, a gel-like structure member of the calcium silicate hydrate (C–S–H) family, and sodium carbonate (Figure 9) [54]. Plombierite, named Tobermorite 14 Å, with the chemical composition Ca_5_Si_6_O_16_(OH)_2_·8H_2_O, is the most hydrated phase of the C–S–H group [55]. The SEM images show large plates of calcium hydroxide crystals after three minutes (Figure 10a) and a typical needles network of C–S–H after three hours (Figure 10b). These results confirm the previous hypothesis concerning the mechanism of coagulation of the soil by the addition of a calcium-containing mineral: the formation of C–S–H as an anti-plasticizer. Moreover, the process involves a coupling of portlandite dissolution and C–S–H precipitation, which delays the coagulation process providing time for processing the earth pastes in the fluid state.

**Figure 11:**
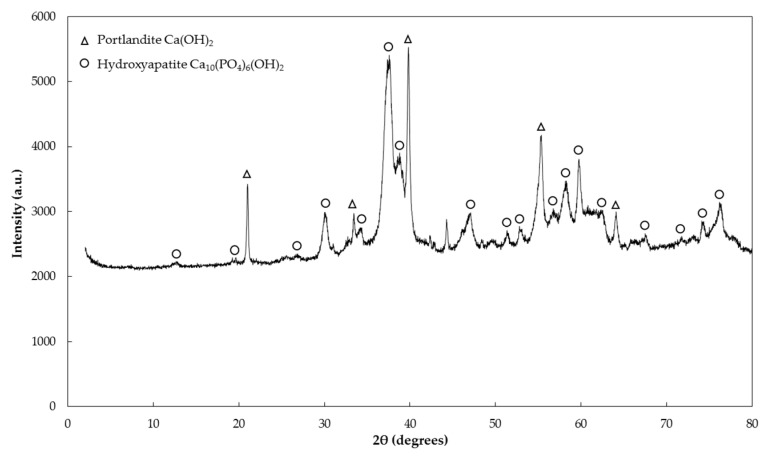
X-ray diffraction pattern of NaHMP solution and Ca(OH)_2_ at 3 h mixing dried at 60 °C and 50 mbar.

When NaHMP is used as a dispersant, we observe the rapid formation of hydroxyapatite [56,57]. This phosphate mineral precipitates in the suspension and, thus, removes the phosphate from the solution (Figure 11).

## 5. Conclusions

The strategies that have been tested are promising and allow for the development of a process to cast a clay-based concrete as easily as a cement bound concrete. By modifying the clay properties with inorganic additives and a material that contains calcium, it is possible to deflocculate and flocculate clays during the casting process.

The use of sodium silicate or sodium hexametaphosphate as dispersant led to a strong deflocculation of clay particles by creating repulsive forces between particles. The yield stress of the clay paste is then considerably reduced, and good workability is obtained, which is required to pour the material in the formwork.

Furthermore, we showed that the addition of calcium hydroxide can reflocculate clay particles through a slow dissolution that releases calcium ions into the interstitial solution, which macroscopically brings the earth material back to its initial behavior. The released calcium ions lead to C–S–H precipitating with the silicate dispersant or hydroxyapatite precipitating with the phosphate based dispersant. This precipitation cancels the plasticizing effect. In this process, in contrast to most earth stabilized products, C–S–H precipitation is used not as a binding agent but as an anti-plasticizer that removes the inorganic dispersant additives. This is confirmed by the fact that the critical strain of the reflocculated clay suspension does not exhibit a behavior characteristic of a suspension controlled by C–S–H interaction.

This study presents a breakthrough compared to previous studies on castable earth. Instead of adding 5 to 10% of lime or cement, 0.2 to 0.5 wt % of lime are added as the amount of calcium introduced is there to counteract the action of the plasticizer used in very small proportion compared to earth material. This has fundamental consequences on the carbon footprint that is reduced by a factor 10 compared to the current practice.

However, further work on the development of a performant self-compacting clay concrete must be performed. This process preserves the initial material but does not improve its internal cohesion and resistance. As a consequence, additional removal of the water is still required to achieve higher strength.

## Figures and Tables

**Table 1:** Chemical composition of the earth studied by X-ray fluorescence analysis. Values are given in mass %.

SiO_2_	TiO_2_	Al_2_O_3_	Fe_2_O_3_	MnO	MgO	CaO	Na_2_O	K_2_O	P_2_O_5_	Cr_2_O_3_	Ignition Loss
65.8	1.2	14.9	7.4	0.1	1.1	1.1	0.5	2.4	0.3	0.02	5.02

**Table 2:** Mineralogical composition of the earth studied by X-ray diffraction.

Quartz	Smectite	Illite	Kaolinite	Muscovite	Plagioclase/Albite	Others	Total
40.9	23.8	5.2	7.5	7.7	7.1	7.7	99.9

**Table 3:** ζ-potential values for the reference earth, the earth prepared with 2% PCE, the earth prepared with 0.2% NaHMP and the earth prepared with 0.3% NaSil.

Parameter	Reference	2% PCE	0.2% NaHMP	0.3% NaSil
Average ζ (mV)	−4.6	−4.5	−13.4	−9.7
SD (mV)	±0.1	±0.1	±0.4	±0.2
